# Plasmonically Enhanced Superradiance of Broken-Symmetry Diamond Color Center Arrays Inside Core-Shell Nanoresonators

**DOI:** 10.3390/nano12030352

**Published:** 2022-01-22

**Authors:** Dávid Vass, András Szenes, Balázs Bánhelyi, Mária Csete

**Affiliations:** 1 Department of Optics and Quantum Electronics, University of Szeged, Dóm tér 9, 6720 Szeged, Hungary; Vass.David.Imre@stud.u-szeged.hu (D.V.); Szenes.Andras.Laszlo@stud.u-szeged.hu (A.S.); 2 Department of Computational Optimization, University of Szeged, Árpád tér 2, 6720 Szeged, Hungary; banhelyi@inf.u-szeged.hu

**Keywords:** superradiance, plasmonics, nanoresonator, broken-symmetry, cooperativity

## Abstract

Superradiance was demonstrated in broken-symmetry arrays of SiV diamond color centers embedded into concave plasmonic nanoresonators. The coupled configurations, including the diamond-silver (bare) and diamond-silver-diamond (coated) nanoresonators’ geometry parameters as well as the emitters’ azimuthal orientation and distance from the metal, were numerically optimized. An objective function consisting of the total fluorescence enhancement multiplied by the corrected emission quantum efficiency was used to design nanoresonators that promote superradiance. A larger total fluorescence enhancement was achieved via a larger number of emitters in both geometries, in coated spherical and in bare ellipsoidal nanoresonators. The superradiance performance was better in the case of a smaller number of emitters in bare spherical and coated ellipsoidal nanoresonators and in the case of a larger number of emitters in coated spherical and bare ellipsoidal nanoresonators. Ellipsoidal geometry is advantageous independent of composition and seeding. The configurations optimal for non-cooperative fluorescence enhancement and superradiance are coincidental. A radiative rate enhancement proportional to the number of emitters was found in wide spectral regions; therefore, superradiance implies *N*-fold enhancements coexist at excitation and emission. In ellipsoidal nanoresonators, the better superradiance achieved via a smaller quality-factor is accompanied by larger frequency pulling.

## 1. Introduction

Single-photon sources (SPS) are crucial in quantum cryptography and metrology; among them, different diamond color centers are favorable [1,2,3]. The promising properties of nitrogen vacancy (NV) diamond color center are its stability, unique spectral characteristics and the achievable spin-polarization entanglement. This color center can be efficiently excited via optical fibers [4]. The spin ensembles of NVs have a spontaneous emission (SE) limited long relaxation time [5], which makes them suitable for quantum key distribution setups [6] and nanoscaled magnetometers’ development as well [7]. Another promising SPS is the silicon vacancy (SiV) color center in diamond, which is particularly interesting due to its strong and extremely narrow zero phonon line near 737 nm at room temperature [8,9].

The spontaneous emission of single-photon sources can be significantly improved due to the increased local density of optical states (LDOS) in the vicinity of plasmonic nanoresonators (NRs), which enables the lifetime of emitters to be decreased via the Purcell effect [10]. The degrees of freedom in plasmonic spectral engineering and the achievable LDOS enhancement are strongly geometry-dependent. 

Hollow plasmonic nanoresonators, such as circular waveguides, periodic patterns of spherical rings and apertures, allow for multiple bands engineering with controlled polarization sensitivity that is advantageous also in emission enhancement [11,12,13].

The particular advantage of nanoresonators with a dielectric-metal and bimetal core-shell composition is that the plasmon resonance frequency can be widely varied due to the distance-dependent coupling between the plasmons localized on neighboring shell interfaces [14,15]. Metal-semiconductor core-shell particles can promote plasmon-induced resonant energy transfer and hot electron injection as well [16].

However, the interaction of emitters and plasmonic nanoparticles can result in enhanced absorptance, transparency and strong-coupling as well; the spectral response depends on the molecular oscillators’ strength and on the exciton transition width [17].

The plasmonic enhancement of spontaneous emission was demonstrated for NV and SiV diamond color centers as well [18,19,20,21,22,23,24,25,26,27,28]. The previously used plasmonic nanoresonators include convex nano-objects, e.g., singlets and doublets of nanospheres [18,19], nanorods [20,21], nanowires [22,23,24], patch-antennas [25] and concave core-shell nanoparticles [26], as well as periodic arrays of convex nanospheres and concave plasmonic nanoapertures [19,27,28]. Further emission improvement is possible by exploiting cooperative phenomena, e.g., superradiance (SR) that makes it possible to develop extremely fast photonic circuit elements smaller than the resonant wavelength, which is crucial in many quantum information processing applications.

Superradiance, originally examined by Dicke, is a cooperative phenomenon between emitters confined into a volume smaller than the wavelength [29]. When *N* emitters interact with and cooperatively radiate through a common electromagnetic field, the emission is accompanied by angular correlation, and the intensity of the emitted light is proportional to *N*^2^ since the transitional probabilities are proportional to the square of the matrix elements of the interaction energy [29]. It was experimentally demonstrated that the timescale of SR is determined by the enhanced radiative rate [30]. It was revealed that the large inversion explains why the system can superradiate [31]. The SR usually results in a burst that is accompanied by various phenomena, such as frequency chirp, beating and polarization fluctuations. In the case of SR, both the sub- and superradiant fields are nearly uniform on a subwavelength scale, which promotes the development of a cooperative Dicke-state that is a quantum system of the *N* two-level atoms facilitating coherent emission due to their matched phase [32]. In the most elementary Dicke system comprising two interacting atoms or ions, SR dominates the radiation in the distance region of (100 nm, 1000 nm) [33]. The coherent light illumination of coherent atoms (nominated as Bose–Einstein condensate) results in a simultaneous superradiant emission both of light and atoms [34].

Plasmonic antennas themselves are good SR candidates due to their large dipole moments. SR of plasmonic origin was reported for Ag clusters dispersed in a glass host, which was proposed to generate short pulses [35]. Superradiance was observed in plasmonic antenna arrays, where each individual antenna acting as a dipolar emitter was coupled cooperatively due to the interaction with the common EM-field [36]. The linewidth broadening in arrays of plasmonic nanorods was attributed to the radiation reaction term enhancement, which is proportional to the number of dipoles, and manifests itself in an *N*-fold increase of the extinction cross-sections’ full-width at half maximum (*FWHM*).

Single-photon sources can also compose superradiant systems, but instead of the usual zero g^(2)^ second-order correlation function, a larger-than-unity value was experimentally demonstrated for NV color center multitudes in diamond nanocrystals [37]. An NV color center spin ensemble in diamond embedded into a lumped element resonator resulted in a superradiant pulse a trillion times faster than the intrinsic decay due to the resonance with the microwave cavity mode [38]. Photon-mediated coupling of two SiV color centers in a diamond nanocavity resulted in spectrally resolvable radiation peaks, indicating distinguishable superradiant and subradiant states [39]. 

The appearance of a central peak in the g^(2)^ second-order correlation function was demonstrated and explained by the increased probability of photon coincidences due to the superradiant emission rate [40].

It is hard to generate cooperative phenomena of emitters in free-space experimentally. The direct dipole–dipole interactions and the small deviations between emitter frequencies can easily hinder the coherent field’s build-up. It is a straightforward idea that the direct dipole–dipole interaction could be controlled if the emitters could be coupled through a common plasmonic field. Therefore, it is expected that tailored nanoresonators are capable of enhancing cooperative phenomena plasmonically. A well-known barrier is, when individual emitters interact with lossy plasmonic nanoresonators, the quenching can prevent SE enhancement. In the case of small emitter distances from the metal, the quenching can be efficiently suppressed, and the emitter ensembles can be strongly coupled to higher order plasmonic modes [41]. However, in this scenario, the interaction occurs between each individual emitter and a pseudomode; therefore, it is a non-cooperative phenomenon.

The elementary examples of plasmonic cooperative and Dicke systems were two-emitters coupled via waveguides. It was demonstrated that the wedge is suited for a donor–acceptor energy transfer, whereas the metallic channel is appropriate for superradiance [42]. The SE of multiple emitters embedded into plasmonic nanocavities can be improved due to the cooperative energy transfer (CET) as well, which fundamentally differs from the Dicke superradiance. In CET, the quantum emitters primarily interact with the plasmon field rather than with each other. The enhanced radiation originates from the plasmonic antenna. Therefore, its spectrum is governed by the plasmonic resonance quality factor and the antenna efficiency. The advantage of plasmon resonance line-shape retaining is the insensitivity to the frequency difference between emitters. When emitters of random location and orientation are coupled to the three dipolar modes of a plasmonic nanosphere, both the radiative and non-radiative decay rates scale with the *N* number of emitters, whereas three-fold enhancement in quantum efficiency can be achieved at larger distances [43,44]. The universal cooperative *Purcell factor* was also derived, which includes the plasmonic enhancement of individual emitters’ decay rates and the acceleration of the ensemble cooperative emission due to plasmonic correlations between them [45,46]. Inside a plasmonic nanocavity resulting in a nearly constant LDOS, the CET is proportional to the number of excited emitters, and its efficiency can be dynamically tuned in a wide range by varying the excitation power [47]. A unified theory was also developed for the compensation of loss accompanying the cooperative energy transfer and spasing [48]. The impact of the emitter orientation and distance was revealed when the coherent response in CET and spasing was described [48]. It was shown that the distance corresponding to the quenching threshold can be decreased by increasing the molecules’ concentration in the active medium of spasers [49].

Many types of plasmon-mediated superradiance phenomena are reported so far. In the case of superradiance, both the position and orientation of emitters may have a significant impact. The intensity enhancement could vary by 10 orders of magnitude when the lossy nanoresonator size is tuned. In the case of a lossy silver nanoresonator, elevational orientation is preferred in terms of intensity enhancement, whereas the most efficient decay rate improvement can be achieved in a radial orientation [50]. Both for lossy and epsilon-near-zero (ENZ) nanoresonators, the existence of optimal distance was revealed in the elevational orientation. Similar 5-fold and 2.5-fold intensity and 14-fold and 4-fold radiative rate enhancement was achieved via elevational emitters around a silver and ENZ nanoresonator, respectively. It was shown that in the optimal elevation configuration, one single bright plasmonic mode is excited, and the emitters synchronously interact with it, while the Purcell-enhanced superradiance can result in a fast burst [51].

Among quantum coherent collective states, the single-photon sources’ superradiance (SPS-SR) was also inspected in plasmonic environments. 

SPS-SR was electrically driven in an array of a ZnPC molecular chain aligned in a tunnel junction acting as a plasmonic nanocavity [52]. It was shown that nanocavity plasmons result in decay rate modification and intensity enhancement. Moreover, the single-photon character might be preserved due to the intrinsic coherence having an intermolecular origin of dipole–dipole coupling.

It is a straightforward idea to enhance the emission of indistinguishable, symmetrically ordered emitters with a spherical nanoparticle. In the case of spherical metal nanoshells acting as concave nanoresonators around an embedded emitter ensemble, the resonance tunability can be exploited, and the strongly and uniformly enhanced EM-field inside the core can promote superradiance [46,47,53,54].

In our previous studies, we have demonstrated how the superradiance of SiV color centers can be plasmonically boosted when they are arranged inside various core-shell nanoresonators in symmetrical arrays [55,56]. The advantages of ellipsoidal geometry, diamond-silver bare composition and a larger number of dipoles have been demonstrated.

In our present study, nanoresonators of diamond-silver core-shell and diamond-silver-diamond multi-shell compositions were modelled, and the superradiance of broken-symmetry emitter arrays embedded into the core was numerically investigated. The configuration of superradiant systems was optimized to achieve a maximal cooperative fluorescence enhancement, utilizing the fact that the bad-cavity characteristic is advantageous for superradiance, and the capabilities of different configurations were compared.

## 2. Materials and Methods

Our previous studies have revealed that there is a trade-off between the total fluorescence enhancement (*P_x_ factor*, defined as the product of the radiative rate enhancements at the excitation (*δR_ex_*) and emission (*δR_em_*)) and the antenna efficiency that is corrected with the intrinsic SiV color center quantum efficiency at the emission (*cQE*). These studies proved that optimization realized by using the objective function of *P_x_* and P_x_*cQE = *P_x_* × *cQE* product promotes to design efficient non-cooperative fluorescent and superradiant configurations, respectively [26,55,56]. Accordingly, we have selected the P_x_*cQE quantity as the objective function for the present numerical optimization. The robustness of a method relying on this objective function is due to the relationship between the nanoresonator quality factor (*Q-factor* that is proportional to the *Purcell factor*) and superradiance, considering that bad-cavity characteristics allow it to achieve a maximal radiative rate, meaning on the level of superradiance [55,56].

The presented results originate from theoretical calculations performed by numerical methods. A finite element numerical method was applied by using COMSOL Multiphysics and an in-house developed optimization algorithm [56,57]. Similar to our previous studies, configurations based on nanoresonators of spherical and ellipsoidal geometry, diamond-metal core-shell (bare) and diamond-metal-diamond multishell (coated) composition embedding four and six SiV color centers were optimized to achieve superradiance by using the P_x_*cQE objective function that has to be maximized [55]. The release of the symmetry restriction is the novelty of the present study, namely, the four and six SiV color centers were modelled as dipolar emitters by arranging electric point sources into rectangular and hexagonal arrays of a central but non-perfect symmetry (Section 3.1 and Section 3.2, Supplementary Material Figure S1).

In addition to the geometrical parameters (radius of the core (*R*), thickness of the silver shell (*t*)), the dipolar emitters’ distance from the metal shell (*d*) and azimuthal orientation (*γ*) were also optimized, whereas the outer diamond layer was fixed (*l* = 25 nm). The additional degree of freedom due to *γ* variation allows for the optimization of broken-symmetry arrays. At a small emitter-metal distance, the *Purcell factor* rapidly increases, but the quantum efficiency approaches zero that causes the quenching phenomenon [10]. 

Increasing the distance from the metal, these quantities tend to intermediate values that enable the radiative rate to be enhanced. The effect of quenching can be neglected in the present study since the emitter-metal distances are large enough in the optimized systems (please see Supplementary Material Figure S2 and Table S1).

By assuming that no quenching occurs at the inspected emitter-metal distance and that the emitters do not have an intrinsic loss, the *Purcell factor*, quantum efficiency (*QE*) and the radiative rate enhancement (*δR*) were computed as follows:

(1) $$Purcell\;factor=\frac{P^{radiative}+P^{nonradiative}}{P_{0}^{radiative}},$$

(2) $$QE=\frac{P^{radiative}}{P^{radiative}+P^{nonradiative}},$$

(3) $$\delta R=Purcell\;factor\times QE=\frac{P^{radiative}}{P_{0}^{radiative}}.$$

Here, the *P^radiative^* and *P_0_^radiative^* were determined by integrating the power outflow throughout a virtual, closed spherical surface around the seeded nanoresonators and bare arrays, respectively, whereas the *P^nonradiative^* was determined based on the resistive heating of the nanoresonators. Finally, the quantum efficiency was corrected by considering the intrinsic quantum efficiency (*QE_0_*~10%) of the SiV color center:

(4) $$cQE=\frac{\delta R}{Purcell\;factor+\frac{1-QE_{0}}{QE_{0}}}.$$

The optical response (*Purcell factor*, quantum efficiency and radiative rate enhancement) was studied as a function of wavelength, and the near-field and far-field phenomena were inspected at the excitation (*λ_ex_* = 532 nm) and emission (*λ_em_* = 737 nm) wavelengths of SiV color centers, similar to our previous works (Section 3.1 and Section 3.2) [20,21,26,55,56]. The distribution of the normalized **E**-field on the equatorial plane and of the charge both on the outer and inner dielectric-metal interfaces was determined. The far-field lobes were extracted by using the Stratton–Chu formula [58]. The superradiance performance in the optimized broken-symmetry array-seeded nanoresonators was inspected by comparing the optical response of the complete array to the optical response of composing individual emitters (Section 3.3). Namely, one single emitter coupled to an identical nanoresonator served as the reference system for optimized nanoresonators seeded by four emitters, whereas the responses of emitters in the two distinguishable positions were averaged in nanoresonators of a geometry identical with those seeded by six emitters. The cooperative fluorescence was primarily qualified by considering the radiative rate enhancement ratios with respect to the reference system (*rδR_ex_*, *rδR_em_*) at the excitation and emission wavelength.

Then, the SR performance was qualified by comparing the *rδR_ex_*, *rδR_em_*, *rcQE*, *rP_x_* and rP_x_*cQE ratios, and finally, a conclusion was made based on their ∑*rX* sum calculated as:

(5) $$\sum rX=\frac{\frac{r\delta R_{ex}}{N}+\frac{r\delta R_{em}}{N}+rcQE+\frac{rP_{x}}{N^{2}}+\frac{rP_{x}{}^{*}cQE}{N^{2}}}{5},$$

where *N* is the number of dipoles in the complete array. The *FWHM* of the *Purcell factor* and radiative rate enhancement spectra of active multiple emitter-seeded nanoresonators were compared to the *FWHM* of the extinction (*ecs*) and scattering (*scs*) cross-section spectra of passive nanoresonators with identical geometry in order to analyze the modification of cavity *Q-factor* (that was calculated as the resonance frequency divided by the corresponding *FWHM*) due to seeding and to inspect the coherence-related linewidth modification.

The frequency pulling (Δ*f*, evaluated by comparing the detunings of maxima in the cold and seeded cavity spectra with respect to the SiV emission wavelength, then the difference between the *ecs* and the *δR* peak’s relative positions was divided by the Δ*λ_δR_* detuning of the δ*R* peak) was also determined to check the expected lower *Q-factor*–larger pulling–better SR performance correlation [59].

The characteristics of the fluorescence and superradiance were determined to conclude about the possible advantages of spherical and ellipsoidal geometry for different seedings (four (four)) as well as for bare and coated NR composition. Comparative statements are presented about the ellipsoidal and spherical geometry.

The distance dependence of the *Purcell factor* as well as the accumulated charge on the dielectric-metal shell interfaces (Section 3.3, please see Supplementary Material Figure S3) were studied to inspect the indistinguishability of emitters. The *Purcell factor* tendencies were inspected in more detail by increasing the coupled dipole’s distance in 5 nm intervals consisting of the optimal one, and the values were normalized to the maximum taken in the inspected intervals. For single emitters outside the quenching regions, the usual ordinary and extraordinary *Purcell factor* and charge tendencies are the inverse proportionality and proportionality to the distance. This is held for the SiV color centers in the inspected concave nanoresonators as well, except at the emission wavelength in bare and coated spherical nanoresonators. (The optical responses through small distances are provided in Supplementary Material Figure S2).

The origin of different branches was determined by studying the cross-polarization effects at different distances (Section 3.3), the degree of which was calculated as the arctangent of the ratio of the E_x_ and E_y_ component of the **E**-field at the excitation wavelength [60]. The cross-polarization angle was determined for the reference and for the completely seeded systems as well. The objective function was compared for optimized configurations embedding SiV color centers of the random and equal phase (Section 3.3). The final conclusion about the optimized configuration was made by considering the *P_x_*, P_x_*cQE, Q-factor, ∑*rX* sum, Δ*λ* detuning and Δ*f* frequency pulling and Δ*f*/Δ*λ_δ__R_* frequency detuning ratios.

All data regarding the geometry (Table S1, Figure S1), the effect of quenching at a small distance outside the inspected ones (Figure S2), the distance dependence of the normalized accumulated charge on the dielectric-metal shell interfaces (Figure S3), optical response (Table S2), ratios of different quantities with respect to the corresponding reference system (Table S3), ratios normalized by the number of emitters (Table S4) and nanoresonator qualifications (Table S5) are presented in the Supplementary Material.

## 3. Results

The core radius of spherical nanocavities took on values in between the short and long axis of their ellipsoidal counterparts, independent of the number of dipoles and the nanoresonator composition. Accordingly, the core volume of four (six) SiV color center seeded bare ellipsoidal nanoresonators was ~7.9 (~5.7) times smaller, and the coated ellipsoidal nanoresonators had ~1.7 (~1.6) times smaller core volume. The metal shell was ~1.3 (1.5) times and ~2.3 (~2.3) times thicker in bare and coated ellipsoidal nanoresonators than in counterpart spherical nanoresonators. The generalized aspect ratio (*GAR* = *R*/(*R* + *t*)) corresponding to the long axis of ellipsoidal nanoresonators was commensurate with, whereas the *GAR* corresponding to the short axis was 1.6 (1.6) times and 1.9 (1.9) times smaller than the *GAR* of bare and coated spherical nanoresonators seeded by four (six) SiV color centers, respectively. The dipole distance was ~6.1 (4.8) times and ~2.3 (1.7) times smaller in bare and coated ellipsoidal nanoresonators than in their spherical counterparts, in accordance with the smaller core. The differences between spherical and ellipsoidal NRs were considerably larger in the case of bare composition in the core volume and in the dipole distance.

The optical responses were similar in bare and coated nanoresonators of spherical and ellipsoidal geometry, independent of the number of dipoles. 

The fundamental difference was that in spherical nanoresonators, the excitation and emission configurations were equivalent, whereas in ellipsoidal NRs, the optical responses differed due to the different modes excitable along their oscillation direction in the two inspected configurations inside elongated concave NRs.

### 3.1. Optical Responses of Bare Ellipsoidal and Spherical Nanoresonators

**Figure 1:**
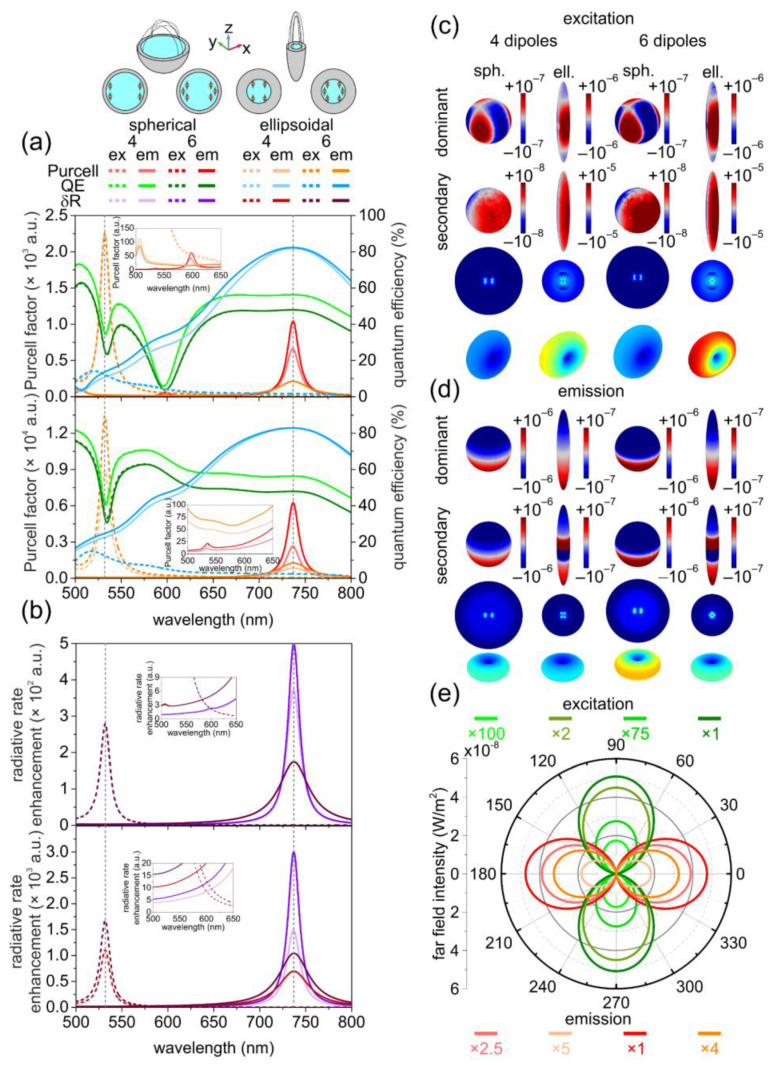
Optical response of bare spherical and ellipsoidal core-shell nanoresonators. Wavelength dependent (**a**) *Purcell factor* and quantum efficiency; (**b**) radiative rate enhancement in the case of the reference (upper) and completely seeded (lower) system. (**c**,**d**) Surface charge densities on the inner metal surface (dominant (top) and secondary (bottom)); near-field distribution in the equatorial plane and far-field distribution of coupled nanoresonators at the (**c**) excitation and (**d**) emission wavelength. (**e**) Angular distribution of the far-field emitted power outflow at both wavelengths.

In spherical bare nanoresonators, a global maximum arose both in the *Purcell factor* and in the *δR* spectrum at the emission wavelength of the SiV color center (737 nm), which was accompanied by a tiny local maximum at the excitation wavelength (532 nm) in the *Purcell factor* spectrum (Figure 1a,b). In the quantum efficiency, there was a narrow local minimum at the excitation, whereas a broad local maximum was observable at the emission. In contrast, in ellipsoidal bare nanoresonators, the decay phenomena were enhanced both at the excitation and emission due to the large single *Purcell factor* peak appearing at the excitation wavelength and to the small single *Purcell factor* peak developing at the emission wavelength in the corresponding configurations. There was a tiny local enhancement at the excitation wavelength in the *Purcell factor* in the emission configuration as well. In the quantum efficiency of bare ellipsoidal NRs, a small and a large global maximum arose close to the excitation and at the emission wavelength in corresponding configurations, respectively. However, compared to the spherical NRs, the reached *QE* value was considerably smaller at the excitation, whereas it was significantly larger at the emission in bare ellipsoidal nanoresonators, independent of seeding. Similarly to the achieved *Purcell factor*, the *δR* values were significantly larger at excitation, whereas they were considerably smaller at emission in ellipsoidal bare nanoresonators for both inspected seedings.

#### 3.1.1. Quantitative Analyses of the Optical Responses of Bare Nanoresonators at Excitation

The quantitative analysis shows that in spherical bare nanoresonators, the achieved 11 (18) *Purcell factor* and the reached 41% (36%) quantum efficiency corresponded to a 5-fold (6-fold) excitation rate enhancement. In comparison, the 9 × 10^3^ (10^4^) *Purcell factor* and 13% (13%) *QE* resulted in orders of magnitude larger 10^3^ (2 × 10^3^) excitation rate enhancement in ellipsoidal bare nanoresonators.

The *Purcell factor* was slightly larger when either a spherical or ellipsoidal bare nanoresonator was seeded by six dipoles that correlated with the larger amount of accumulated charge. These relationships are governed by the distance dependence of the *Purcell factor* and accumulated charge in bare nanoresonators. The tendency for both seedings is the ordinary inverse proportionality with the distance in spherical bare NRs, whereas it is extraordinary for both quantities in bare ellipsoidal NRs (more details in Section 3.3, and see Figures S2a,b and S3a in the Supplementary Material).

A significant ~26.2 times (30.0 times) larger amount of charges accumulated at the dielectric-metal interfaces of ellipsoidal bare nanoresonators than on their spherical counterparts (Figure 1c, Table S2). The far-field lobes at excitation were significantly larger in ellipsoidal bare nanoresonators, which correlated with the orders of magnitude larger excitation enhancement (Figure 1e, Table S2).

#### 3.1.2. Quantitative Analyses of the Optical Responses of Bare Nanoresonators at Emission

The *Purcell factor* took on a 3 × 10^3^ (6 × 10^3^) value, which was accompanied by 56% (48%) *QE*, and corresponded to a 2 × 10^3^-fold (3 × 10^3^-fold) emission rate enhancement in spherical bare nanoresonators. In comparison, the 8 × 10^2^ (10^3^) *Purcell factor* and the accompanying 82% (82%) quantum efficiency made it possible to achieve a 7 × 10^2^-fold (10^3^-fold) emission rate enhancement in ellipsoidal bare nanoresonators. 

The *Purcell factor* values were larger in nanoresonators seeded by six dipoles that correlated with the larger amount of accumulated charge, independent of the nanoresonator geometry. Considering the extraordinary and ordinary distance-dependence of the *Purcell factor* and charge that was similar for the two inspected seedings, the increase can be explained by cooperative effects and different geometrical parameters in six-emitters-seeded spherical and ellipsoidal bare nanoresonators, respectively (more details in Section 3.3, and see Figures S2a,b and S3a in the Supplementary Material). 

The extraordinary *Purcell* factor and charge tendencies were inherited from the single SiV seeded bare spherical nanoresonators but were more pronounced.

The slightly non-monotonous distance dependence of the *Purcell factor* for four SiV seeded ellipsoidal nanoresonator did not have an impact on the relationship between two different seedings.

The amount of accumulated charge was ~2.44 times (2.15 times) smaller on ellipsoidal bare nanoresonators in their optimal emission configurations (Figure 1d, Table S2). According to the smaller emission rate enhancements, the far-field lobes of ellipsoidal bare nanoresonators were considerably smaller compared to their spherical counterpart. As a result, the relationship between far-field lobes was reversed with respect to excitation (Figure 1e, Table S2).

### 3.2. Optical Responses of Coated Ellipsoidal and Spherical Nanoresonators

**Figure 2:**
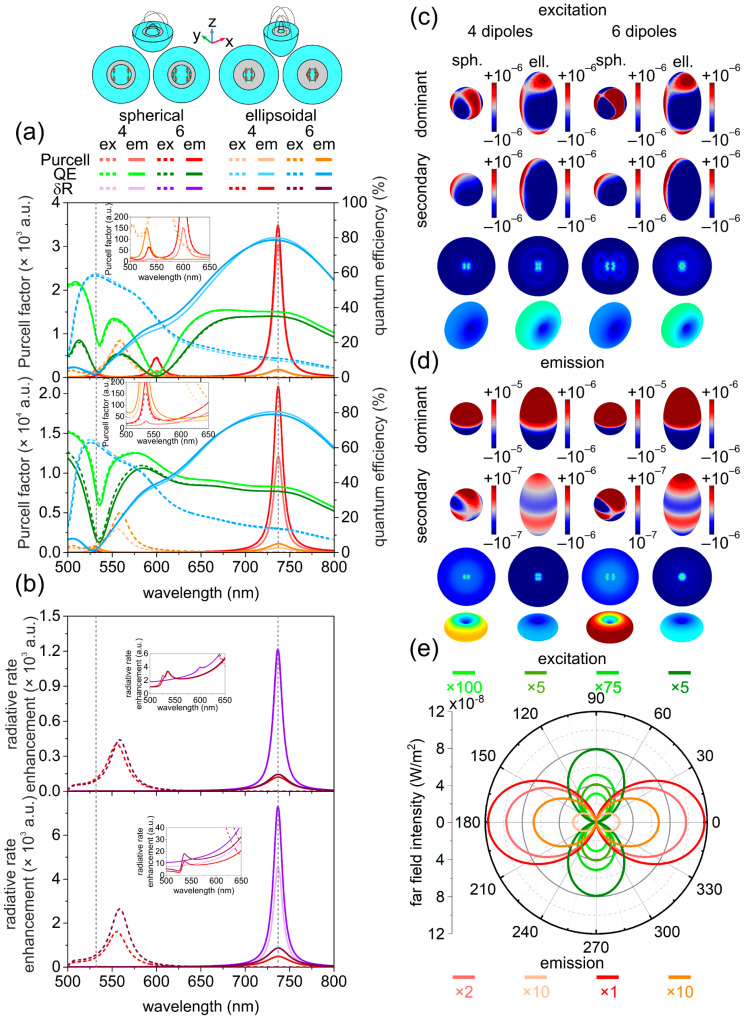
Optical response of coated spherical and ellipsoidal core-shell nanoresonators. Wavelength dependent (**a**) *Purcell factor* and quantum efficiency; (**b**) radiative rate enhancement in the case of the reference (upper) and completely seeded (lower) system. (**c**,**d**) Surface charge densities on the outer metal surface (dominant (top) and secondary (bottom)) near-field distribution in the equatorial plane and far-field distribution of coupled nanoresonators at the (**c**) excitation and (**d**) emission wavelength. (**e**) Angular distribution of the far-field emitted power outflow.

The advantage of coating on spherical nanoresonators was that two well-defined peaks arose in the *Purcell factor* spectrum—one tiny peak at the excitation and another larger maximum at the emission wavelength (Figure 2a).

In the quantum efficiency, there was a narrow local minimum at excitation, whereas a plateau was observable at emission. As a result, the *δR* spectrum was similar to that of the bare type NR spectrum, namely, one single peak appeared at emission (Figure 2b).

The coating on the ellipsoidal nanoresonators had similar effects, namely, a larger number of peaks arose in the *Purcell factor* spectrum in both configurations. In the excitation configuration, one peak appeared below and another above the excitation wavelength (near 506 nm and slightly above 550 nm); therefore, only the latter peak could contribute to the SiV fluorescence improvement. In the emission configuration, one tiny peak appeared at the excitation and another at the emission wavelength, but only the latter could contribute noticeably to the SiV color centers’ fluorescence enhancement due to their orientation. The achieved *Purcell factor* values became significantly smaller at excitation and noticeably smaller at emission compared to those in bare ellipsoidal counterpart nanoresonators. In the quantum efficiency spectrum, a relatively larger and a relatively smaller global maximum arose close to the excitation and at the emission wavelength, respectively. Accordingly, the achieved *QE* values were more commensurate at the two inspected wavelengths compared to bare ellipsoidal nanoresonators. As a result, in the *δR* spectrum, a global radiative rate enhancement maximum appeared close to excitation and at emission, which was smaller compared to the values reached in bare ellipsoidal nanoresonators in the corresponding configurations. The global *δR* maximum at emission was accompanied by a tiny radiative rate enhancement peak at excitation in the emission configuration of coated ellipsoidal NRs.

There were additional peaks close to and at the excitation wavelength in the *δR* spectrum in the excitation and emission configurations of ellipsoidal coated nanoresonators compared to their spherical counterparts. The reached *QE* was larger both at excitation and at emission in ellipsoidal coated nanoresonators. As a result, in coated nanoresonators, the spherical–ellipsoidal *QE* relationship was reversed at excitation, whereas it was preserved at the emission compared to bare nanoresonators.

The achieved *Purcell factor* and *δR* values were larger at excitation, whereas they were smaller at emission in ellipsoidal coated nanoresonators compared to their spherical counterparts, similar to bare nanoresonators.

#### 3.2.1. Quantitative Analyses of the Optical Responses of Coated Nanoresonators at Excitation

The quantitative analysis shows that in spherical coated nanoresonators, the achieved 22 (10^2^) *Purcell factor* and reached 37% (10%) quantum efficiency corresponded to an 8-fold (12-fold) excitation rate enhancement. The *QE* was smaller, whereas the *Purcell factor* and excitation rate enhancement were larger than in their bare counterpart. 

In comparison, the 7 × 10^2^ (9 × 10^2^) *Purcell factor* and 64% (62%) *QE* corresponded to a one order of magnitude larger 4 × 10^2^ (6 × 10^2^) excitation rate enhancement in ellipsoidal coated nanoresonators. The *QE* was larger, whereas the *Purcell factor* and excitation rate enhancement were smaller than in their bare counterpart. 

These features indicate that the coating promotes the SiV color center excitation phenomenon in spherical NRs, whereas it is not advantageous in ellipsoidal NRs. The *Purcell factor* and accumulated charge values were larger in spherical and ellipsoidal coated nanoresonators when they were seeded by six dipoles.

These relationships were governed by the ordinary distance dependence of the *Purcell factor* and accumulated charge in coated spherical and ellipsoidal nanoresonators at excitation (more details in Section 3.3 and see Figures S2c,d and S3b in the Supplementary Material). As a result, at excitation, a ~4.3 times (1.6 times) larger amount of charges accumulated on the surface of ellipsoidal nanoresonators compared to their spherical counterparts (Figure 2c, Table S2).

The ratio of charges accumulated on spherical and ellipsoidal coated nanoresonators was significantly smaller compared to the ratio of charges accumulated on bare nanoresonators. On coated spherical nanoresonators, there was a larger amount of charge than on their bare counterparts, which correlated with the larger *Purcell factor*. In comparison, in coated ellipsoidal nanoresonators, the smaller amount of charge correlated with the smaller *Purcell factor* compared to bare counterparts. These relationships are held despite the different configuration parameters.

The far-field lobes were considerably larger in ellipsoidal coated nanoresonators, according to the larger excitation rate enhancement (Figure 2e, Table S2). The difference was less pronounced than in the case of bare nanoresonators.

#### 3.2.2. Quantitative Analyses of the Optical Responses of Coated Nanoresonators at Emission

In coated spherical nanoresonators, the achieved 10^4^ (2 × 10^4^) *Purcell factor* and reached 38% (35%) quantum efficiency corresponded to a 5 × 10^3^-fold (8 × 10^3^-fold) emission rate enhancement. The *QE* was smaller, whereas the *Purcell factor* and radiative rate enhancement were larger than in their bare spherical counterparts, whose relationships are analogous to those at excitation. In comparison, the 6 × 10^2^ (10^3^) *Purcell factor* and the accompanying 79% (78%) quantum efficiency made it possible to achieve a 5 × 10^2^ (9 × 10^2^) emission rate enhancement in ellipsoidal coated nanoresonators. The slightly smaller *QE* and *Purcell factor* allowed for a smaller emission rate enhancement than in their bare ellipsoidal counterparts, i.e., only the *QE* relationship was modified compared to excitation. These features indicate that the coating promotes the emission phenomenon in spherical NRs, whereas it is not advantageous in ellipsoidal NRs.

The *Purcell factor* and the accumulated charge values were larger in nanoresonators seeded by six dipoles independent of the nanoresonator geometry, similar to bare nanoresonators. Considering the extraordinary and ordinary distance dependence of the *Purcell factor* and accumulated charge, the increase can be explained by cooperative effects and different geometries in spherical and ellipsoidal coated nanoresonators, respectively (more details in Section 3.3, and see Figures S2c,d and S3b in the Supplementary Material). The extraordinary *Purcell* factor and charge tendencies were inherited from the single SiV seeded coated spherical nanoresonators but were more pronounced.

The amount of accumulated charge was ~7.8 times (7.5 times) smaller on ellipsoidal coated nanoresonators than on their spherical coated counterparts (Figure 2d, Table S2). On coated spherical NRs, a larger *Purcell factor* was achieved compared to their bare counterparts, which correlated with the larger amount of charge despite the different configurations.

On coated ellipsoidal nanoresonators, a smaller *Purcell factor* was achieved compared to their bare counterparts, although the amount of charge was slightly larger at a larger emitter distance in a significantly different elongated geometry.

These examples prove that the ordinary distance—*Purcell factor*—charge amount relationships are held only when one compares similar configurations (similar nanoresonators seeded by four and six SiV), whereas the pronounced difference in geometrical parameters of bare and coated nanoresonators may have an impact on it.

According to the smaller emission rate enhancements, the far-field lobes were significantly smaller in the case of ellipsoidal coated nanoresonators compared to their spherical counterparts. As a result, the relationship between far-field lobes was reversed compared to excitation, similar to bare nanoresonators (Figure 2e, Table S2).

### 3.3. Superradiance Qualification in Bare and Coated Ellipsoidal and Spherical Nanoresonators

#### 3.3.1. Optical Responses of Reference Systems

In the reference system corresponding to bare and coated spherical nanoresonators, there was an additional local maximum in the *Purcell factor* spectrum near 600 nm (Figure 1a and Figure 2a). A reference system-specific global minimum appeared in the quantum efficiency spectrum at the same wavelength.

The cumulative effect of these extrema results was an additional tiny peak in the radiative rate spectrum (Figure 1b and Figure 2b). These extrema originated from nonradiative quadrupolar resonance on the reference system at 600 nm.

In comparison, in the excitation configuration of reference bare (coated) ellipsoidal NRs, a local minimum and maximum developed in the *QE* and *Purcell factor* spectrum below 500 nm (at 506 nm).

In the emission configuration of bare and coated ellipsoidal nanoresonators, a local maximum appeared in the *Purcell factor* spectrum, and a global minimum developed in the quantum efficiency spectrum near 506 nm and 532 nm that were specific and non-specific to the reference system, respectively. Similar to the spherical NR, at and close to these extrema, a tiny additional local maximum appeared in the radiative rate enhancement spectrum in the emission configuration of the reference bare and in both configurations of reference and completely seeded coated ellipsoidal nanoresonators in the inspected interval, respectively.

#### 3.3.2. Spectral Dependence of the SR Performance

**Figure 3:**
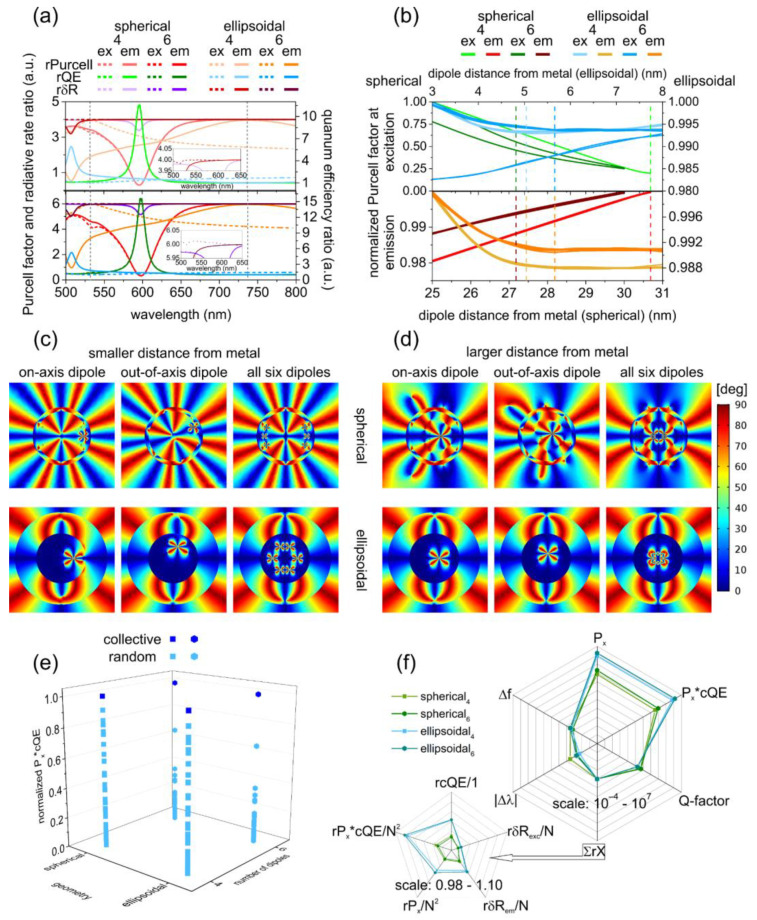
Superradiance of bare spherical and ellipsoidal core-shell nanoresonators. (**a**) Wavelength dependent *Purcell factor*, quantum efficiency and radiative rate enhancement ratios in the case of four (upper) and six (lower) dipoles, with respect to corresponding reference systems (**b**) normalized *Purcell factor* of individual emitters as a function of distance from the metal shell at the excitation (upper) and at the emission (lower) wavelength (data ranges are limited by NR size). Angle distribution of cross-polarization in the case of (**c**) small and (**d**) large dipole distance from metal (magnified to ensure that the apparent core sizes are identical in the case of spherical and ellipsoidal nanoresonators). (**e**) Comparison of the P_x_*cQE achieved via random and synchronized SiV color centers. (**f**) Comparison of SR performance in spherical and ellipsoidal bare nanoresonators.

**Figure 4:**
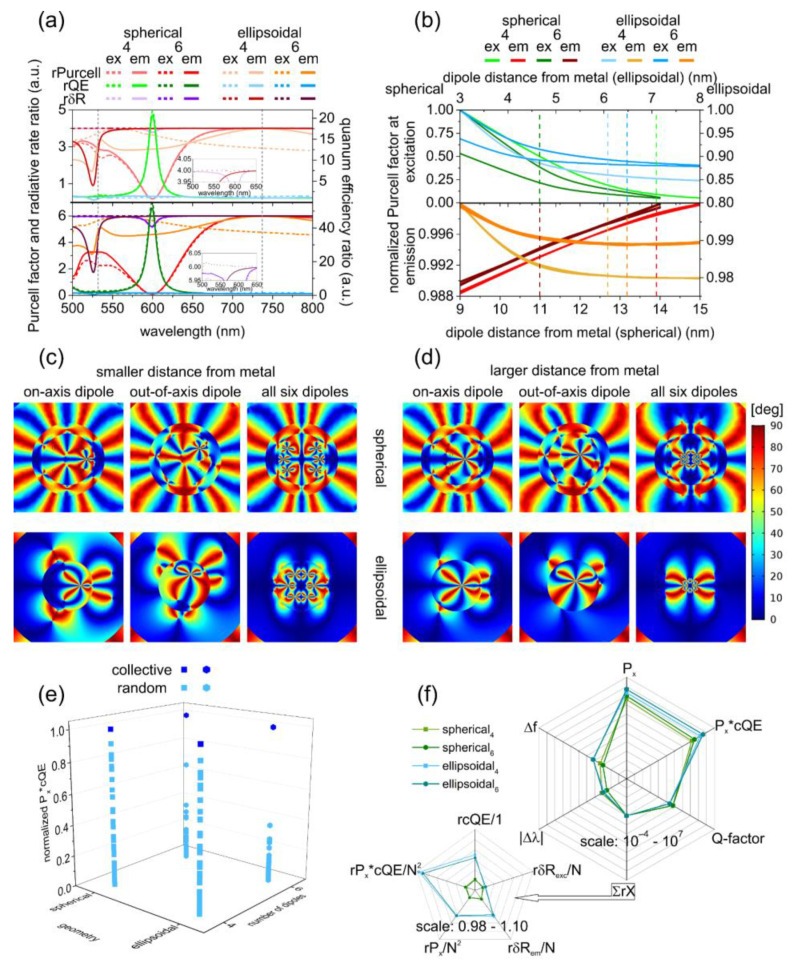
Superradiance of coated spherical and ellipsoidal core-shell nanoresonators. (**a**) Wavelength dependent *Purcell factor*, quantum efficiency and radiative rate enhancement ratios in the case of four (upper) and six (lower) dipoles with respect to corresponding reference systems (**b**) normalized *Purcell factor* of individual emitters as a function of distance from the metal shell at the excitation (upper) and at the emission (lower) wavelength (data ranges are limited by NR size). Angle distribution of cross-polarization in the case of a (**c**) small and (**d**) large dipole distance from metal (magnified to ensure that the core sizes are identical in the case of spherical and ellipsoidal nanoresonators). (**e**) Comparison of the P_x_*cQE achieved via random and synchronized SiV color centers. (**f**) Comparison of SR performance in spherical and ellipsoidal bare nanoresonators.

The optimized nanoresonators ensured SR throughout wide spectral intervals, e.g., in the radiative rate enhancement spectrum normalized by the *δR* spectrum of the reference system, there were only a few locations, where the normalized value was smaller than the wide-band constant ratio of four and six (Figure 3a and Figure 4a). These locations are related to the above-described extrema in the *δR* spectra of the corresponding reference systems.

In spherical nanoresonators, the global minimum in the normalized *rPurcell* spectrum and the global maximum in the normalized *rQE* spectrum corresponded to a quadrupolar resonance. This appeared at 596 nm (598 nm) and 600 nm (600 nm) in both configurations of the bare and coated spherical nanoresonators seeded by four (six) emitters, respectively. This was accompanied by a global minimum in the *rδR* spectrum at the same wavelength.

In the excitation configuration of bare ellipsoidal nanoresonators caused by a small local minimum in *rPurcell* *factor* below and a global minimum in *rQE* close to the excitation wavelength, a tiny minimum appeared in their overlapping interval (510 nm) in the *rδR* spectrum. In the emission configurations of bare ellipsoidal nanoresonators seeded by four (six) emitters, a global minimum appeared in the normalized *rPurcell* *factor* spectrum and a global maximum appeared in the normalized *rQE* spectrum at 506 nm (506 nm). This corresponded to a pronounced global minimum in the *rδR* spectrum at the same spectral location. In comparison, in the excitation configuration of coated ellipsoidal nanoresonators, a tiny *rPurcell factor* spectrum peak and *rQE* spectrum dip appeared nearby at 506 nm wavelength, whereas global *rPurcell* and *rcQE* minima appeared nearby at 526 nm in the emission configuration.

As a result, in the *rδR* spectrum of coated ellipsoidal nanoresonators, a tiny minimum appeared nearby at 506 nm in the excitation configuration, whereas a pronounced global minimum developed at 526 nm in the emission configuration. 

This global minimum in the *rδR* spectrum in the case of four (six) dipoles originated from the almost equal radiative rate enhancement values in the reference and completely seeded systems in ellipsoidal coated nanoresonators. The forward shift of the *rδR* global minima in the emission configuration of coated ellipsoidal nanoresonators with respect to those on their bare counterparts was in accordance with the spectral effect of the coating.

Although the ratio of the *Purcell factor*, *QE* and *δR* with respect to the reference system (*rPurcell, rQE, rδR*) was smaller in spherical nanoresonators both at the excitation and emission wavelengths, they also overrode the threshold of superradiance, independent of their composition. That means, due to *N* emitters’ interaction that cooperatively radiate through a common plasmonic field, the intensity of the emitted light is proportional to *N*^2^.

#### 3.3.3. Evaluation of Non-Cooperative and Cooperative Responses and SR Performance

When the non-cooperative fluorescence was qualified based on the product of the radiative rate enhancement ratios at the excitation and emission wavelengths (*rPx = rδR_ex_* × *rδR_em_*), in bare spherical NRs, smaller seeding became advantageous, whereas in coated spherical NRs, the non-cooperative fluorescence rate enhancement was independent of seeding. A larger *N* was favorable in bare ellipsoidal NRs, and a smaller *N* was preferred in coated ellipsoidal NRs. The same relationships were true for the FOM = P_x_*cQE qualifying the cooperative fluorescence, except that the FOM became better in the more-seeded coated spherical nanoresonator.

Despite the weaker fluorescence enhancement at emission, both the product of the radiative rate enhancements, namely the *P_x_* total fluorescence enhancement and the derived P_x_*cQE objective function, were two orders and one order of magnitude larger in bare and coated ellipsoidal nanoresonators than in their spherical counterparts, respectively. These results indicate a stronger capability both for non-cooperative and cooperative fluorescence enhancement in ellipsoidal geometry.

The ∑*rX* quantity determined based on the (*rcQE, rδR_ex_, rδR_em_*, *rP_x_*, rP_x_*cQE) ratios showed a better superradiance performance for smaller number of emitters in bare spherical and coated ellipsoidal nanoresonators and for larger number of emitters in coated spherical and bare ellipsoidal nanoresonators. The ∑*rX* quantity was larger in ellipsoidal nanoresonators independent of seeding and composition, which revealed a better superradiance performance in the case of elongated geometry.

#### 3.3.4. Evaluation of Indistinguishability

In the case of four dipoles in the distance-dependent, normalized *Purcell factor*, a 4-fold degeneracy was observable at both wavelengths independent of composition and geometry. In the case of six dipoles, a 6-fold degeneracy held at emission, but two branches were identifiable at excitation independent of geometry and composition—one with a 2-fold and the other with a 4-fold degeneracy that corresponded to dipoles located on the x-axis and relocated above and below it, respectively (Figure 3b and Figure 4b).

In spherical bare nanoresonators, in the case of four dipoles at excitation, monotonous *Purcell factor* decrease, whereas at emission, monotonous *Purcell factor* increase was observable (Figure 3b). In the case of six dipoles, the normalized *Purcell factor* exhibited an exponential decrease at excitation, with a smaller and larger rate for the relocated and on-axis emitters, respectively. A uniform *Purcell factor* increase was observable at emission when the distance from the metal was increased.

In ellipsoidal bare nanoresonators, the normalized *Purcell factor* exhibited a non-monotonous distance dependence (exponential decrease was followed by a slow increase) in the case of four dipoles at both wavelengths. In the case of six dipoles, the normalized *Purcell factor* exhibited a non-monotonous distance dependence and monotonous increase at excitation for emitters located on the axis and relocated with respect to it, respectively. A uniform *Purcell factor* decrease was observable at the emission for both seedings; the degree of indistinguishability was similar, but the tendency was reversed compared to the distance dependency for emitters in spherical NRs.

The distance dependence was ordinary at excitation and extraordinary at emission in spherical bare nanoresonators, whereas it was extraordinary at excitation and ordinary at emission in ellipsoidal bare NRs, except for the slightly non-monotonous tendency at the emission for four SiV centers.

In spherical coated nanoresonators, in the case of four dipoles, the *Purcell factor* branches showed an exponential decrease at excitation and a monotonous increase at emission in a larger *Purcell factor* interval, which were more rapid at both wavelengths compared to tendencies in bare counterpart NRs (Figure 4b).

In the case of six dipoles, the normalized *Purcell factor* exhibited an exponential decrease at excitation for both branches, which was faster and slower for the emitters on the axis and relocated with respect to it, respectively. A uniform *Purcell factor* increase was observable at emission when the distance from the metal was increased. In the case of spherical nanoresonators, the most significant modification due to coating was in the slope of the *Purcell factor* at excitation. 

The achieved *Purcell factor* values were less and more commensurate for the two different seedings in coated spherical nanoresonators compared to their bare counterparts at excitation and emission, respectively.

In comparison, in ellipsoidal coated NRs, the normalized *Purcell factor* exhibited a uniform rapid exponential decrease in the case of four dipoles at both wavelengths. In the case of six dipoles, the normalized *Purcell factor* exhibited a faster and slower exponential decrease at excitation for the dipoles on the axis and relocated with respect to it, respectively.

A uniform exponential *Purcell factor* decrease was observable at emission; the degree of indistinguishability was similar, but the tendency was reversed compared to the distance dependency for emitters in spherical nanoresonators, similar to their bare counterparts.

In the case of ellipsoidal nanoresonators, the coating resulted in tendency modifications. For small-seeding, the distance dependence became monotonous, with an exponential decrease at both wavelengths.

For large-seeding, the tendency became a similar exponential decrease for both branches at excitation, whereas the exponentially decreasing tendency was preserved at emission. The distance dependence was ordinary in the excitation configuration and extraordinary in the emission configuration of spherical coated nanoresonators, whereas it was ordinary in both configurations of ellipsoidal coated nanoresonators.

The existence of two branches originated from the different degrees of cross-polarization at different locations. At small distances from the metal, the emitters’ behavior was non-collective; as a consequence, there was a difference in between the angles qualifying the cross-polarization (Figure 3c and Figure 4c).

By increasing the distance from the metal, the difference between the emitters diminished, and they started to oscillate collectively. As a result, the degree of cross-polarization decreased and the branches became indistinguishable (Figure 3d and Figure 4d).

#### 3.3.5. Comparison of Nanoresonator Capabilities and Supported Modes

Based on the larger *FWHM* of *ecs*, cold ellipsoidal cavities possessed a significantly smaller *Q-factor*, which was advantageous in SR performance improvement. Moreover, all spectral peaks exhibited a significantly larger *FWHM* seeded in ellipsoidal nanoresonators.

Detuning both of the *Purcell factor* and *δR* peaks was smaller in less-seeded ellipsoidal nanoresonators, independent of composition. Linewidth narrowing with respect to the passive counterparts occurred in bare type spherical nanoresonators in radiative rate enhancement and in bare ellipsoidal nanoresonators in *Purcell factor*, independent of the number of color centers as well as in more-seeded bare spherical NRs in the *Purcell factor*. In comparison, linewidth narrowing did not occur in spherical coated nanoresonators, whereas in ellipsoidal coated nanoresonators, linewidth narrowing was achieved both in *δR* radiative rate enhancement and in *Purcell factor* spectral peaks, independent of the number of SiV color centers. Considering that linewidth narrowing in the *Purcell factor* indicates a *Q-factor* increase with respect to cold nanocavities, whereas in *δR* it indicates better coherence; the former is not advantageous, but the latter is favorable in superradiance achievement and application, respectively.

The bare spherical nanoresonators showed better coherence accompanied by a smaller *Q-factor* when they were less seeded, whereas they exhibited a slightly larger *Q-factor* when they were more seeded. In coated ellipsoidal nanoresonators, the better coherence was achieved despite the simultaneously larger *Q-factor* after seeding. However, the achieved *Purcell factor FWHM* was larger and the related *Q-factor* was smaller when the inspected coated ellipsoidal geometry was compared either to bare or coated spherical nanoresonators.

The ellipsoidal nanoresonators exhibited a larger Δ*f* frequency pulling towards the emitter, in accordance with theoretical predictions based on *Q*-factor relationships [59]. In bare ellipsoidal NRs seeded by four (six) color centers, the smaller *Q-factor* was accompanied by a considerably (moderately) larger frequency pulling towards the SiV emission, which allowed for a better superradiance performance, in accordance with intuitive expectations. Both seedings were more advantageous in the SR achievement than their spherical counterparts. In comparison, in coated ellipsoidal nanoresonators, a considerably larger frequency pulling accompanied the smaller *Q-factors* independent of seeding, which allowed for a better superradiance performance; accordingly, they were similarly more advantageous in the SR achievement.

A comparison of randomly and collectively oscillating systems shows that random systems were more similar in the case of a smaller number of emitters. The random phase emitters in bare and coated spherical nanoresonators better approximated the collective nature than in the ellipsoidal counterpart nanoresonators in the case of a larger emitter number.

The most significant P_x_*cQE improvement due to synchronization was achieved in the case of a larger number of dipoles in coated ellipsoidal geometry (Figure 3e and Figure 4e).

The time evolution of the charge distribution uncovered that less-radiative hexapolar and radiative dipolar modes appeared on the inner and outer shell surfaces of spherical and ellipsoidal nanoresonators at the excitation wavelength, except on the outer surface of bare ellipsoidal NRs, where only hexapolar modes developed.

The radiative dipolar modes were accompanied by hexapolar (quadrupolar) modes on the inner and outer shell surfaces of coated spherical (both ellipsoidal) nanoresonators at the emission wavelength, whereas in bare spherical nanoresonators, only dipolar modes developed (Figure 1c,d and Figure 2c,d).

The larger emission rate enhancement correlated with the larger dipole fraction compared to excitation in spherical nanoresonators, independent of composition. In bare (coated) ellipsoidal nanoresonators, the larger excitation rate correlated with the larger fraction of dipoles on the inner surface (absence of quadrupolar and presence of hexapolar modes beside dipolar modes that were relatively more dominant on the outer surface). The larger fraction of dipolar modes on the inner and outer surface explains why bare and coated ellipsoidal nanoresonators resulted in a significantly and considerably larger excitation rate enhancement with respect to their spherical counterparts, respectively. Similarly, the appearance of a significant and considerable fraction of quadrupolar modes on the inner and outer surface both of bare and coated ellipsoidal nanoresonators explains the smaller emission rate enhancement compared to their spherical counterparts.

## 4. Conclusions

The advantages of a (smaller) larger number of color centers in (bare) coated spherical and (coated) bare ellipsoidal composition, as well as of the ellipsoidal geometry in the achievement of larger total fluorescence enhancement (*P_x_ factor*) and higher FOM (P_x_*cQE) were demonstrated. It was shown that the far-field lobes were larger for a larger number of dipoles in all nanoresonators, for coated spherical and bare ellipsoidal composition and for ellipsoidal geometry, both at the excitation and emission wavelengths.

In bare spherical nanoresonators, the conclusion regarding the advantage of a smaller number of dipoles is preserved; in the coated spherical composition, the larger seeding becomes advantageous in FOM, revealing a cooperative fluorescence even though it is not dominant in non-cooperative emission (*P_x_ factor*).

The possibility to achieve superradiance by embedding SiV diamond color centers into concave plasmonic nanoresonators was demonstrated. It was shown that broken-symmetry of the emitter arrays does not prevent superradiance when multiple SiV centers are embedded into rectangular and hexagonal patterns. 

Based on the summarized ratios (∑*rX*), to achieve better superradiance performance, a smaller number of emitters in bare spherical and coated ellipsoidal NRs and a larger number of emitters in coated spherical and bare ellipsoidal NRs is preferable. By considering non-cooperative fluorescence and SR performance, the same was concluded regarding the advantage of ellipsoidal geometry, and the larger number of SiV color centers in ellipsoidal nanoresonators of bare composition, as well as the smaller number of SiV color centers in coated ellipsoidal NRs. 

Moreover, bare spherical and coated ellipsoidal configurations exhibit linewidth narrowing with respect to the passive NRs in the radiative rate enhancement as well, which indicates better coherence.

The *Q-factor* is smaller for a smaller number of emitters (except the bare ellipsoidal NRs) for bare composition and for ellipsoidal geometry. The frequency pulling is larger for a larger number of emitters in spherical nanoresonators and for a smaller number of emitters in ellipsoidal nanoresonators and for a coated composition (except the more-seeded spherical nanoresonators) and for ellipsoidal geometry. The frequency pulling–*Q-factor*–SR performance correlation holds for all ellipsoidal-spherical nanoresonator’s comparison. Comparison of nanoresonators with different geometry shows that larger *P_x_* and P_x_*cQE as well as ∑*rX* is achievable with smaller *Q-factor*, larger Δ*f* as well as smaller Δ*λ* (except the more seeded cases) in ellipsoidal nanoresonators than in their spherical counterparts (Figure 3f and Figure 4f). Based on the summarized ratios (∑*rX*), coated ellipsoidal nanoresonators seeded by four emitters can be proposed for SR performance.

These studies revealed that the radiative rate is enhanced proportionally with the number of emitters throughout a wide spectral region; therefore, the total fluorescence rate enhancement scales with ~*N*^2^ (Figure 3a and Figure 4a). This is due to the collective radiative modes that develop on each plasmonic nanoresonator seeded by multiple emitters. The radiative rate enhancement correlates with the amount of charge accompanying the dipolar modes on the complete shell of each nanoresonator. Demonstrated correlations with dipolar modes on the inner surface of bare nanoresonators and on the outer surface of coated NRs uncovered the preferred localization area of the dominant radiative modes. Exceptional spectral intervals are those where non-radiative quadrupolar modes develop on the nanoresonators seeded by one emitter considered as reference systems, since these modes cause narrow gaps in SR.

The SR emission can be transferred to the far-field via enhanced radiative modes (Figure 1e and Figure 2e). However, the SR is sensitive to the distance from the metal, and when a larger number of dipoles is embedded into the nanoresonator, the indistinguishability is destroyed, caused by depolarization effects (Figure 3b–d and Figure 4b–d). 

Considering that depolarization strongly depends on the emitter-metal distance, controlled implantation of SiV color centers is crucial. Alternatively, homogeneously distributed color centers could be selectively enhanced, when a monolayer of nanoresonators is excited through a lattice mode of proper symmetry. Spherical nanoresonators can be arranged into a hexagonal monolayer, whereas the ellipsoidal nanoresonators can be replaced by cylindrical diamond cavities coated by thin metal layers that can be fabricated in hexagonal and rectangular lattices via e-beam lithography.

## Data Availability

Data are contained within the article or Supplementary Materials.

## Supplementary Materials

The following supporting information can be downloaded at: https://www.mdpi.com/article/10.3390/nano12030352/s1, Figure S1: Schematics of the optimized coupled system types; Figure S2: Optical responses normalized to unity as a function of emitter distance from metal; Figure S3: Electric charge accumulated on the optimized core-shell surface as a function of emitter distance from the metal; Table S1: Geometric parameters of optimized core-shell nanoresonators; Table S2: Optical responses of optimized systems; Table S3: Enhancements compared to reference systems; Table S4: Enhancements compared to reference systems that are normalized by the emitter number; Table S5: Spectral properties of the optimized core-shell nanoresonators.
