# Supplementary material for: Plasmonically Enhanced Superradiance of Broken-Symmetry Diamond Color Center Arrays Inside Core-Shell Nanoresonators
Source: Nanomaterials (Basel). 2022 Jan 22;12(3):352. doi: 10.3390/nano12030352 (PMC8840258; doi:10.3390/nano12030352)
Supplement: Supplementary file 1 [file nanomaterials-12-00352-s001.zip › nanomaterials-1541640-supplementary.pdf]

Supplementary Material

Plasmonically enhanced superradiance of broken-symmetry diamond color center arrays inside core-shell nanoresonators

*Dávid Vass, András Szenes, Balázs Bánhelyi and Mária Csete**

*mcsete@physx.u-szeged.hu

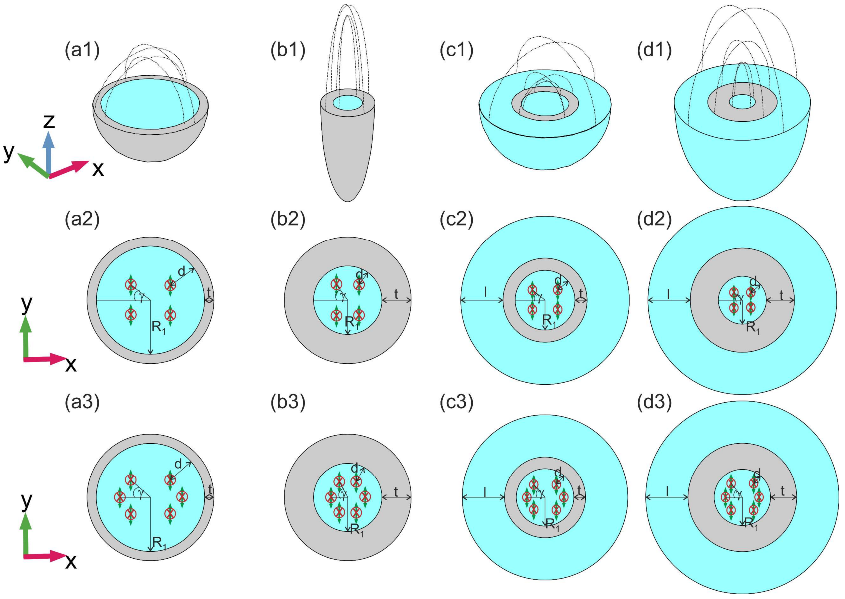

Figure S1. Schematics of the optimized coupled system types: (a-d/1) 3D view, and (a-d/2, a-d/3) x-y plane cross-section of the nanoresonators seeded by (a-d/2) 4 and (a-d/3) 6 emitters in case of (a/1-3, b/1-3) bare, (c/1-3, d/1-3) coated and (a & c/1-3) spherical, (b&d/1-3) ellipsoidal geometry.

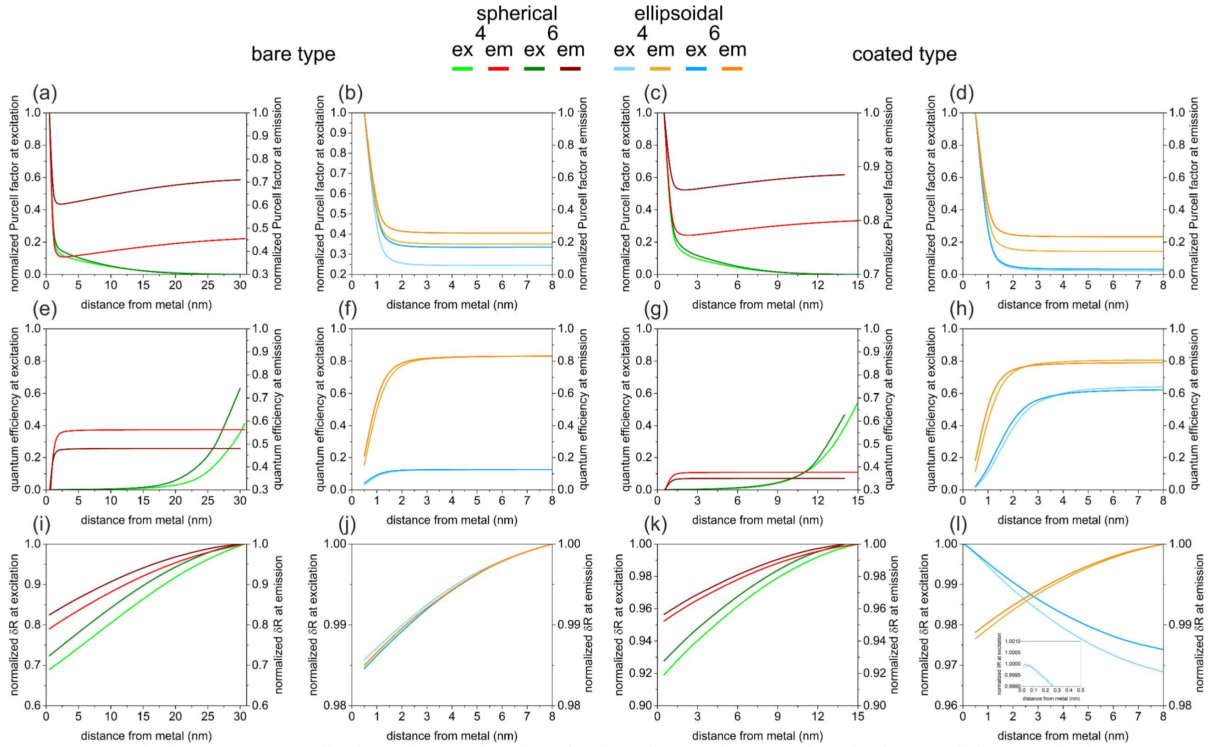

Figure S2. Optical responses normalized to unity as a function of emitter distance from metal. (a-d) The Purcell factor, (e-h) the quantum efficiency and (i-l) the radiative rate enhancement in case of (a, b, e, f, i, j) bare and (c, d, g, h, k, l) coated type, nanoresonators of (a, c, e, g, i, k) spherical, (b, d, f, h, j, l) ellipsoidal geometry.

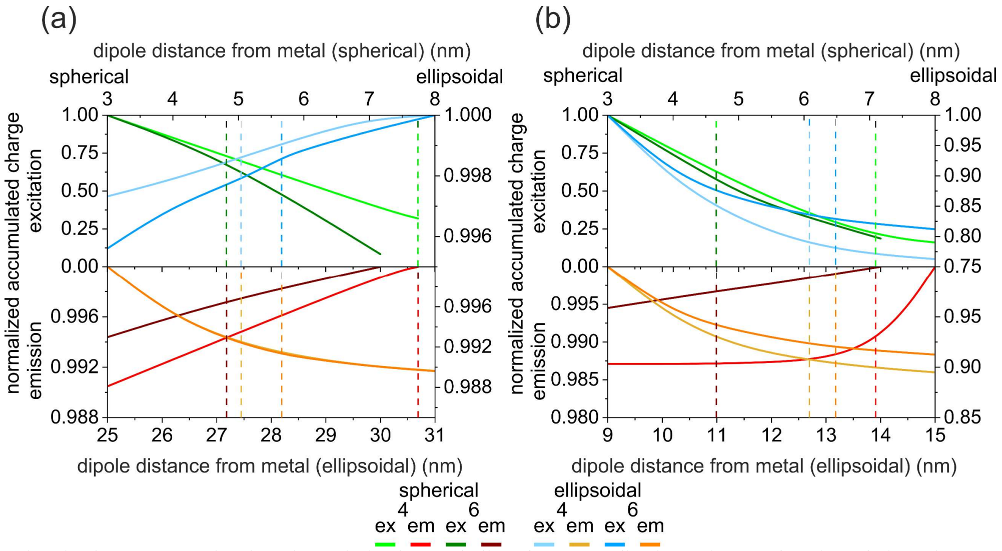

Figure S3. Electric charge accumulated on the optimized core-shell surface normalized to unity as a function of SiV color centers distance from the metal. (a) bare and (b) coated nanoresonators, at the excitation (upper) and at the emission (lower) wavelength. Each curve is presented in a dipole distance interval limited by the size of the nanoresonator.

Colors indicate, when certain quantity is larger in case of

4	6	number of emitters
bare	coated	type of nanoresonator
spherical	ellipsoidal	geometry of nanoresonator

geometry	bare_4		coated_4		bare_6		coated_6	
	spherical	ellipsoidal	spherical	ellipsoidal	spherical	ellipsoidal	spherical	ellipsoidal
R_1 (nm)	38.05	10	17.67	10	34.2	10	17	10
R_2 (nm)	38.05	69.97	17.67	32.47	34.2	70.02	17	31.65
t (nm)	6.61	8.54	7.17	16.78	5.82	8.55	6.94	16.08
d (nm)	30.69	5.04	13.91	6.08	27.18	5.66	10.99	6.48
GAR_1	0.85	0.54	0.71	0.37	0.85	0.54	0.71	0.38
GAR_2	0.85	0.89	0.71	0.66	0.85	0.89	0.71	0.66
γ (°)	14.28	50.67	23.73	53.8	24.94	68.68	36.7	61.94

Table S1. Geometric parameters of optimized core-shell nanoresonators. R_1 and R_2 are the half short and long axes, respectively; t is the metal shell thickness, d is the emitter distance from the metal shell. GAR_1 and GAR_2 is the generalized aspect ratio corresponding to the long and short axes, respectively, defined as $GAR=R/(R+t)$, γ is the azimuthal angle of emitters measured from the x-axis.

**optical response**	bare_4		coated_4		bare_6		coated_6	
	spherical	ellipsoidal	spherical	ellipsoidal	spherical	ellipsoidal	spherical	ellipsoidal
QE_{ex} (%)	41.48	12.5	36.96	63.45	36.27	12.51	9.91	61.87
$Purcell_{ex}$	11.16	9043.64	22.31	685.1	17.75	13621.61	122.81	901.02
δR_{ex} (a.u.)	4.63	1130.27	8.25	434.68	6.44	1703.79	12.17	557.44
$charge_{ex}$ (C)	3.64E-21	9.55E-20	1.11E-20	4.79E-20	4.77E-21	1.43E-19	4.16E-20	6.84E-20
$far\text{-}field_{ex}$ *lobe max.* (W/m^2)	1.73E-10	2.25E-08	3.10E-10	8.22E-09	3.66E-10	5.09E-08	6.84E-10	1.58E-08
QE_{em} (%)	56.15	81.86	37.69	79.25	47.94	82.29	35.04	78.26
$Purcell_{em}$	2682.22	841.71	12576.22	605.42	6410.09	1256.1	21756.12	1106.39
δR_{em} (a.u.)	1506.1	689.04	4740.23	479.78	3073	1033.59	7622.26	865.81
$charge_{em}$ (C)	5.64E-20	2.31E-20	3.07E-19	3.94E-20	1.09E-19	3.46E-20	4.96E-19	6.61E-20
$far\text{-}field_{em}$ *lobe max.* (W/m^2)	1.56E-08	3.64E-09	4.87E-08	2.52E-09	4.73E-08	8.15E-09	1.17E-07	6.78E-09
P_x factor	6.97E+03	7.79E+05	3.91E+04	2.09E+05	1.98E+04	1.76E+06	9.27E+04	4.83E+05
P_xcQE	3.92E+03	6.38E+05	1.47E+04	1.65E+05	9.48E+03	1.45E+06	3.25E+04	3.78E+05

Table S2. Optical responses of optimized systems. *Purcell* factor is the total decay rate enhancement, δR is the radiative decay rate enhancement of N emitters, QE is the antenna efficiency calculated as $QE=\delta R/Purcell$, *charge* is the electric charge accumulated on the metal shell, *far-field lobe max.* is the maximal power radiated to the far-field at one meter from the structure, "ex" and "em" subscript refers to the excitation and emission configuration, respectively; P_x *factor* is the product of δR_{ex} and δR_{em} qualifying the non-cooperative fluorescence enhancement and P_xcQE is the P_x *factor* weighted by the corrected antenna efficiency (the objective function), that promotes to design superradiant systems.

**ratio**	bare_4		coated_4		bare_6		coated_6	
	spherical	ellipsoidal	spherical	ellipsoidal	spherical	ellipsoidal	spherical	ellipsoidal
rcQE	1.01	1.04	1	1.05	1.01	1.04	1	1.04
rδR_{ex}	3.98	4	3.99	4	5.97	6.01	5.97	6.01
rδR_{em}	4.04	4.13	4	4.17	6.04	6.21	6.01	6.24
rP_x	16.07	16.5	15.97	16.7	36.06	37.33	35.92	37.5
rP_xcQE	16.23	17.2	15.97	17.53	36.32	38.91	36.01	39.16

Table S3. Enhancements compared to reference systems: $rcQE$ is the corrected antenna efficiency ratio at the emission, $r\delta R_{ex/em}$ is the radiative rate enhancement ratio at excitation/emission, rP_x is the total non-cooperative fluorescence enhancement and rP_xcQE is the weighted fluorescence enhancement ratio.

normalized ratio	bare_4		coated_4		bare_6		coated_6	
	spherical	ellipsoidal	spherical	ellipsoidal	spherical	ellipsoidal	spherical	ellipsoidal
r$cQE/1$	1.01	1.042	1	1.05	1.007	1.043	1.002	1.044
r$\delta R_{ex}/N$	0.994	1	0.998	1	0.995	1.001	0.996	1.002
r$\delta R_{em}/N$	1.01	1.031	1	1.044	1.007	1.035	1.002	1.04
rP_x/N^2	1.004	1.031	0.998	1.043	1.002	1.037	0.998	1.042
r$P_x cQE/N^2$	1.014	1.075	0.998	1.096	1.009	1.081	1	1.088
ΣrX	1.007	1.036	0.999	1.047	1.004	1.039	1	1.043

Table S4. Enhancements compared to reference systems that are normalized by the emitter number N (or N^2): $rcQE$ is the corrected antenna efficiency ratio at the emission, $r\delta R_{ex/em}$ is the radiative rate enhancement ratio at excitation/emission, rP_x is the total non-cooperative fluorescence enhancement and $rP_x cQE$ is the weighted fluorescence enhancement ratio. ΣrX is the average of the five normalized ratios, quantifying the superradiance performance.

nanoresonator qualification		bare_4		coated_4		bare_6		coated_6	
		spherical	ellipsoidal	spherical	ellipsoidal	spherical	ellipsoidal	spherical	ellipsoidal
ecs	*FWHM* (nm)	17.1	40.35	11.88	32.5	14.57	40.43	11.46	30.15
	Q factor	43.1	18.27	62.05	22.73	50.61	18.24	64.35	24.49
	$\Delta\lambda$ (nm)	-0.01	0.21	0.34	1.79	0.12	0.28	0.12	1.62
scs	*FWHM* (nm)	17.17	40.08	11.81	32.5	14.64	40.16	11.37	30.16
	$\Delta\lambda$ (nm)	0.5	0.59	1.08	2.43	0.55	0.66	0.88	2.25
Purcell	*FWHM* (nm)	17.16	39.92	11.98	32	14.56	39.99	11.51	29.7
	Q factor	42.93	18.47	61.56	23.04	50.62	18.43	63.99	24.82
	$\Delta\lambda$ (nm)	-0.34	0.06	0.28	0.22	-0.03	0.04	-0.13	0.24
δR	*FWHM* (nm)	17.03	40.26	11.9	32.31	14.5	40.34	11.48	29.97
	$\Delta\lambda$ (nm)	-0.33	-0.02	0.13	0.07	-0.04	-0.05	0.03	0.09
Δf (nm)		0.32	0.23	0.21	1.72	0.16	0.33	0.09	1.53
$\Delta f/\Delta\lambda_{\delta R}$		0.97	11.5	1.62	24.57	4	6.6	3	17

Table S5. Spectral properties of the optimized core-shell nanoresonators. *FWHM* denotes the full-width-at-half-maximum, $\Delta\lambda$ is the detuning from 737 nm emission wavelength of extinction (*ecs*), scattering (*scs*) cross-section, Purcell factor (*Purcell*) and radiative rate enhancement (δR) spectra; *Q factor* is the nanoresonator quality factor calculated from *ecs* and *Purcell* spectra; Δf is the frequency pulling of the superradiant system specified in wavelength, $\Delta f/\Delta\lambda_{\delta R}$ to facilitate comparison of pullings for the cold cavity and emitter.
